# The correlation between left atrial appendage morphology and thromboembolic risk in atrial fibrillation

**DOI:** 10.3389/fstro.2025.1619570

**Published:** 2025-12-09

**Authors:** Chengyi Li, Yaoji Wang, Buyun Xu

**Affiliations:** 1 Department of Cardiology, Shaoxing University School of Medicine, Shaoxing, Zhejiang, China; 2 Department of Cardiology, Shaoxing People's Hospital (The First Affiliated Hospital of Shaoxing University), Shaoxing, Zhejiang, China

**Keywords:** atrial fibrillation, left atrial appendage, left atrial appendage thrombus, left atrial appendage morphology, hemodynamics

## Abstract

Atrial fibrillation (AF) is the most common cardiac arrhythmia and a major cause of ischemic stroke. Between 91% and 100% of cardiogenic thrombi are in the left atrial appendage (LAA), and the morphology of the LAA is closely associated with the formation of LAA thrombus (LAAT). This review provides a detailed discussion of the anatomy of the LAA, the epidemiology, and the diagnosis of LAAT. It focuses on analyzing the role of LAA morphology in blood stasis, morphological abnormality, and hypercoagulable states. Accurate evaluation of the morphology of the LAA can assist with risk stratification in patients with AF. The commonly used LAA morphological evaluation indicators must be more comprehensive and objective. Recently, new imaging protocols allow for LA morphological remodeling and fibrosis assessment, which has been demonstrated to correlate with assessing the individual's risks of thromboembolic events and practical imaging of patients with LAAT.

## Highlights

- The morphology of the LAA plays a significant role in blood stasis, structural changes, and hypercoagulable states, which might be associated with thrombus formation.
- More complex LAA morphology correlates with a higher risk of LAAT.
- A comprehensive and objective assessment of LAA morphology is proposed as essential for improving risk stratification and personalizing treatment strategies for AF patients.

## Introduction

Atrial fibrillation (AF) is one of the most common arrhythmias encountered in practice. According to the Global Burden of Disease Study 2019, the estimated prevalence of AF in adults is between 2% and 4%. Fifty million people worldwide are estimated to be affected (Joglar et al., 2024).

Thromboembolic events are a primary complication of AF, with nearly one-quarter of ischemic strokes potentially attributable to AF (Ding et al., 2023). Thromboembolic complications in AF patients arise because AF is associated with multiple thromboembolic risk factors, such as advanced age, hypertension, diabetes, coronary artery disease, and heart failure (Joglar et al., 2024; Kamel et al., 2016; Mac Grory et al., 2022; Reddy et al., 2022). More importantly, AF itself can lead to cardiogenic thrombus formation (Singer et al., 2021), with 91%−100% of these thrombi located in the left atrial appendage (LAA) (Di Biase et al., 2018). LAA thrombus (LAAT) significantly increases the risk of thromboembolism and is strongly correlated with outcomes of thromboembolic events and overall mortality (Heo et al., 2023; Hautmann et al., 2023; Zhang et al., 2024). Current practice guidelines place significant emphasis on assessing the risk of thromboembolic events in AF patients, particularly the risk of stroke, often overlooking the evaluation of the risk factors of LAAT (Joglar et al., 2024). As previously mentioned, not all thromboembolic events in AF patients are caused by cardiogenic thrombi; there may be differences in the prevention and treatment of thromboembolic events depending on the underlying mechanisms (Mac Grory et al., 2022). Conversely, assessing LAAT risk aids in more precise risk stratification, enhancing individualized treatment and research for AF patients.

As a primary source of thrombi in AF patients, the morphology of the LAA is closely related to the formation of LAAT. On the one hand, the morphology of the LAA can serve as a biomarker for atrial myopathy and various complications associated with AF, and it is thus related to LAAT. On the other hand, the morphology of the LAA itself may directly or indirectly contribute to thrombus formation. To better understand the relationship between LAA morphology and LAAT formation in AF patients, this paper will first provide a brief introduction to LAA anatomy, LAAT epidemiology, and LAAT diagnosis; then, a detailed discussion will be presented on the role of LAA morphology in the formation of LAAT and its ability to predict the risk of LAAT.

## LAA anatomy

The LAA exhibits considerable variability in size, shape, and anatomical relationships with surrounding structures (Gonzalez-Casal et al., 2022). It is located laterally to the LA and anterior to the left pulmonary veins, separated by Warthin's ridge. It is positioned superiorly and anteriorly adjacent to the pulmonary artery and inferiorly adjacent to the left atrioventricular groove and the left circumflex branch. The left phrenic nerve may traverse it laterally.

The definitions of the LAA orifice opening vary, including the anatomical definition, the narrowest part of the LAA body, and Warthin's ridge-circumflex branch plane (Fang et al., 2022). Using the ridge-circumflex plane to define the LAA orifice may aid in standardizing the morphological research on the LAA (Hozawa et al., 2018). The shape of the LAA orifice can be classified as oval (68.9%), tubular (10%), triangular (7.7%), teardrop (7.7%), and round (5.7%) (Madaffari et al., 2020). The shape and size of the LAA orifice are influenced by various factors such as LA filling status and heart rhythm (Hozawa et al., 2018; Spencer et al., 2015).

Typically, the body of the LAA extends anteriorly and superiorly, with some patients exhibiting lateral or posterior extension and a few extending to the pericardial sinus. The average length of the LAA body is 45 mm (Madaffari et al., 2020). The endocardial thickness is uneven, and the atrial wall between the pectinate muscles is relatively thin (less than 1 mm). Thicker pectinate muscles seen on imaging might be misdiagnosed as LAAT (Gonzalez-Casal et al., 2022; Madaffari et al., 2020). The morphology of the LAA body is diverse, with multiple lobes possible. In an autopsy study of nearly 500 cases, 54% of patients had two lobes, 23% had three lobes, 20% had one lobe, and 3% had four or more lobes (Madaffari et al., 2020). Various classification methods have been proposed for LAA morphology based on lobes and characteristics. Due to its ability to predict stroke risk effectively and its guiding significance for LAA closure therapy, the classification method proposed by Wang et al. is currently commonly used, including chicken wing, windbag, cactus, and cauliflower types (Di Biase et al., 2012).

In addition to lobes and pectinate muscles, trabeculae are vital anatomical structures within the LAA that influence its function and local hemodynamics. Though often underappreciated in clinical imaging, recent computational studies have demonstrated their significant role in modulating intra-appendage blood flow and shear stress. Vella et al. showed that trabecular architecture affects flow dynamics and thrombogenic potential in AF (Vella et al., 2024). Likewise, Musotto et al. reported that trabeculae contribute to blood stasis by altering shear gradients and vortex formation (Musotto et al., 2024). These findings suggest that trabeculae should not be regarded as minor structural features but as relevant morphological factors impacting thromboembolic risk. Incorporating trabecular analysis may thus enhance the morphological assessment of the LAA.

In summary, the LAA is one of the most variable parts of cardiac anatomy, and it is highly diverse and irregular. Therefore, a unified, comprehensive, and objective description of LAA morphology is currently needed.

## LAAT epidemiology

Due to the often-asymptomatic nature of LAAT before thrombus detachment, LAAT is frequently discovered incidentally during LAAT screening before AF electrical cardioversion or catheter treatment. As a result, the accurate prevalence of LAAT is challenging to estimate. The prevalence of LAAT varies significantly across different reports due to variations in patient characteristics. A meta-analysis focused on non-anticoagulated patients found that the prevalence of LAAT in individuals with non-valvular AF ranges from 5% to 27% (Azzalini et al., 2018). Other related studies have shown that despite receiving guideline-recommended anticoagulant therapy, the prevalence of LAAT remains as high as 2.73% to 7.2% (Mortensen et al., 2021; Pieszko et al., 2024). The studies mentioned above consider trans-esophageal echocardiography (TEE) the gold standard for detecting LAAT. However, due to its semi-invasive nature, large-scale screening is not feasible.

Additionally, these studies mainly included AF patients undergoing evaluation for catheter ablation or cardioversion, which introduces a selection bias. Additionally, the covert nature of LAAT and the invasiveness of TEE make it challenging to conduct prospective studies focused on LAAT as an endpoint. Therefore, current epidemiological research on LAAT primarily relies on cross-sectional or retrospective studies, which can provide prevalence data but need help to provide incidence information, posing a significant challenge for future research on LAAT.

## LAAT diagnosis

TEE is currently the primary method for diagnosing LAAT. With the intraoperative confirmation of LAAT as the gold standard, TEE has a sensitivity of 93%−100% and specificity of 99%−100% for detecting LAAT (Azzalini et al., 2018). TEE can directly assess the presence of thrombus in the LAA and evaluate LAA morphology, blood flow velocity, emptying fraction, contraction ability, and spontaneous echo contrast, all closely related to LAAT risk. Spontaneous echo contrast is mainly considered a “pre-thrombotic state” for LAAT and, in some studies, has even been used as a surrogate marker for LAAT (Wang et al., 2023). Despite its numerous advantages, TEE is semi-invasive, which can lead to poor patient experience and potential complications such as esophageal perforation. Additionally, TEE has some relative contraindications, such as a history of swallowing difficulties, restricted neck mobility, esophageal varices, and coagulation disorders (Puchalski et al., 2019).

Due to the limitations of TEE, guidelines recommend cardiac-enhanced computed tomography (CT) with biphasic scanning as an alternative to TEE for ruling out LAAT before AF cardioversion or catheter treatment (Joglar et al., 2024). Using TEE results as the gold standard, cardiac-enhanced CT has a specificity and sensitivity of 89% and 95%, respectively, with single-phase scanning. In the biphasic CT, specificity and sensitivity improve to 100% and 99% (Yu et al., 2021). However, the significant radiation exposure associated with biphasic CT has led to studies suggesting that radiomics analysis based on single-phase CT images significantly improves the diagnostic accuracy for LAAT (Chun et al., 2021; Li et al., 2023). Nonetheless, current radiomics research has limited sample sizes and numerous influencing factors, such as scanning parameters, equipment, and parameter extraction algorithms, requiring validation (Yip and Aerts, 2016). Compared to TEE, cardiac-enhanced CT offers lower invasiveness, higher patient acceptance, and more comprehensive anatomical information but lacks assessment of LAA function and carries higher radiation risks and potential contrast-related complications.

Current research indicates that the sensitivity and specificity of cardiac MRI in diagnosing LAAT are comparable to those of enhanced CT. Additionally, advancements in new technologies are expected to improve diagnostic accuracy while reducing patient impact (Vira et al., 2019; Orbán et al., 2023). Intracardiac echocardiography provides a close-range and multi-angle examination of the LAA, achieves a 97% concordance with TEE in diagnosing LAAT, and can detect LAATs that TEE might miss (Srivatsa et al., 2014). It is an alternative for patients unsuitable for TEE or cardiac CT (Jingquan et al., 2022). However, due to its highly invasive nature and high cost, intracardiac echocardiography is primarily used as an adjunct tool during catheter-based treatments (de Leon et al., 2023).

## Pathogenesis of LAAT in patients with AF

Although the association between AF and thrombus formation has long been recognized, the mechanisms underlying the formation of LAAT are not fully understood. According to Virchow's triad for thrombus formation—blood stasis, morphological abnormality, and hypercoagulable states—this section will elucidate the mechanisms of LAAT formation and analyze the roles of LAA and LA morphology in this process.

### Blood stasis

Blood stasis is one of the primary mechanisms for thrombus formation in patients with AF (Qureshi et al., 2023). The LAA, with its morphology characterized by a “narrow opening,” “deep body,” and “abundant pectinate muscles,” exhibits particularly pronounced blood stasis. TEE assessing the blood flow velocity within the LAA of patients with paroxysmal AF shows that the flow velocity during AF is only half that during sinus rhythm (Tubeeckx et al., 2024). When the local blood flow velocity within the LAA decreases, especially when the mean flow velocity falls below 20 cm/s, the risk of thrombus formation within the LAA and subsequent stroke increases significantly (Di Biase et al., 2018; Wegner et al., 2022; Cresti and Camara, 2022).

It is noteworthy that, even in sinus rhythm, there are significant differences in blood flow velocity and other indicators of blood stasis between AF and non-AF patients (Li et al., 2022; Bäck et al., 2023). Therefore, in addition to the AF rhythm, various systemic factors (such as age, obesity, diabetes, heart failure, CHA2DS2-VASc score) and local factors (such as LAA function and morphology) may also contribute to blood stasis. Even in non-AF patients, LAA function and morphology are related to the state of blood stasis within the LAA (Bäck et al., 2023). Different studies use various metrics to assess LAA function and morphology. Table 1 summarizes research on the correlation between LAA morphology and blood stasis in AF patients. Table 1 shows that current evaluations of LAA morphology focus on size and traditional LAA morphology classifications. Larger LAA volumes are associated with more severe blood stasis in the LAA. Traditional LAA morphology classifications suggest that chicken wing-type LAA is associated with less severe blood stasis, while cauliflower-type LAA is associated with more severe stasis. Table 1 refers exclusively to clinical studies, thereby excluding simulation (in-silico) and *in-vitro* studies.

**Table 1:** Study on the correlation between LA and LAA morphology and blood stasis in the LAA.

**Researcher**	**Publication year**	**Number of cases**	**Study conclusion**
Mao et al. (2022)	2022	102	Compared to non-wings-type LAA, patients with wings-type LAA have an 8.6-fold increased risk of LAA flow velocity (LAAFV) < 35 cm/s.
Li et al. (2022)	2022	360	The blood flow velocity in wings-type LAA is higher compared to non-wings-type LAA (55 ± 19 cm/s vs. 41 ± 20 cm/s).
Bäck et al. (2023)	2023	131	Compared to wings-type LAA, patients with non-wings-type LAA have a 2.2-fold increased risk of SEC and a lower LAAFV (39.7 ± 18.8 cm/s vs. 51.4 ± 25.1 cm/s).
Fang et al. (2021)	2021	96	Compared to cauliflower-type (52.7 cm/s) and cactus-type LAA (55.3 cm/s), wings-type (73.7 cm/s) and windbag-type LAA (61.9 cm/s) have higher blood flow velocities.
Vella et al. (2021)	2021	194	LAAFV is negatively correlated with the area of the LAA orifice and the depth of the LAA.
Lo Presti et al. (2025)	2025	808	Compared to non-cauliflower-type LAA, cauliflower-type LAA shows poorer contrast agent filling.
Kishima et al. (2015)	2015	130	For each 1 ml/m^2^ increase in LAA volume index, the risk of SEC increases by 5%.
Lee et al. (2015)	2015	408	When the LAA angle is acute, the risk of LAAFV < 20 cm/s increases by 53%.
Petersen et al. (2015)	2015	81	Compared to non-wings-type LAA, wings-type LAA patients have higher LAAFV (49.1 ± 22.0 cm/s vs. 36.2 ± 15.0 cm/s) and a negative correlation with the LAA orifice area.
Buchner and Endemann (2016)	2016	440	For each 1 ml/m^2^ increase in LAA volume index, the risk of LAAFV < 40 cm/s increases by 9%.
Matsumoto et al. (2017)	2017	641	For each 1 ml/m^2^ increase in LAA volume index, the risk of SEC increases by 31%.

LA, Left Atrium; LAA, Left Atrial Appendage; LAAFV, Left Atrial Appendage Flow Velocity; SEC, Spontaneous Echo Contrast.

In addition to LAA morphological characteristics, the position of the LAA relative to the LA and the synchrony of mechanical contractions may also significantly impact the state of blood stasis within the LAA (Mao et al., 2022; Fang et al., 2021). As demonstrated by Vella et al., LAA models were analyzed and compared based on shear strain rate (SSR) and vorticity—two hemodynamic parameters directly associated with thrombogenicity. Their findings indicated that AF-related alterations in contractility and morphology play a principal role in establishing prothrombotic hemodynamic conditions. These altered flow dynamics predispose patients to a higher incidence of ischemic events, in concordance with existing clinical evidence31. This study, thanks to the use of ideal morphologies, allows for the exclusion of the effect of morphology and thus, isolates and investigates the role of contractility alterations (produced by AF) on the thrombotic potential (Vella et al., 2021). Lo Presti et al. employed a single-physics fluid–structure interaction (FSI) model based on smoothed particle hydrodynamics (SPH) to simulate thrombus formation within patient-specific LAA morphologies under AF conditions. Their study revealed that regions closer to the LAA orifice, despite experiencing stronger washout effects that may delay thrombus accumulation, are more susceptible to flow instability due to increased recirculation. This instability could facilitate embolus detachment and consequently elevate the risk of thromboembolic events. These findings highlight the need to understand the dynamic evolution of clot formation and growth in order to more accurately predict thromboembolic risk (Lo Presti et al., 2025).

In summary, blood stasis is one of the critical mechanisms of LA thrombus formation in AF patients. AF rhythm is not the sole cause of blood stasis within the LAA. Systemic factors, as well as the morphology and function of the LA and LAA, are significantly related to the blood stasis state within the LAA. Furthermore, relative blood flow velocity concerning the LAA wall may be more crucial in LA thrombus formation than the “absolute” blood flow velocity within the LAA. Therefore, in some patients, LAA strain and contraction function may be critical assessment indicators.

### Morphological abnormality

#### Endothelial injury

The endothelium consists of a layer of flattened cells lining the inner surfaces of the heart and blood vessels. It plays roles in anti-inflammatory responses, coagulation balance, and regulation of vascular tone. While the association between AF and endothelial dysfunction is recognized, the causal relationship is more complex. In catheter ablation procedures for paroxysmal AF, comparing intracardiac endothelial function markers before and after AF episodes suggests that AF is a crucial contributor to endothelial damage (Ding et al., 2020; Dagenais et al., 2013).

Regardless of the causal relationship between AF and endothelial function, endothelial dysfunction is crucial in AF-related thrombus formation. To exclude systemic factors that could increase circulating endothelial damage markers, such as hypertension, diabetes, and renal dysfunction, researchers compared blood samples from the atrial chamber and peripheral blood, finding significant differences in endothelial function indicators like von Willebrand factor and nitric oxide between different sample sources. Even between LAA and LA samples, concentration gradients were observed, indicating localized endothelial dysfunction in AF patients and an increased risk of local thrombus formation (Xu et al., 2021). Recent human specimen studies show that LAA endothelial function in AF patients is the best predictor of stroke and LAAT risk. At the same time, blood stasis is not an independent risk factor for LAAT and stroke (Maher et al., 2023).

Hemodynamics at the endothelial cell surface is crucial in regulating endothelial function. Recently, Lai et al. demonstrated that fluid shear stress characteristics, including magnitude, direction, and change frequency, influence endothelial function through fluid dynamics (Lai et al., 2024). Since LAA morphology is a decisive factor in hemodynamics (Gimbrone and García-Cardeña, 2016), it can significantly impact LAA endothelial function. However, directly studying the relationship between LAA endothelial function and LAA morphology presents challenges. On the one hand, evaluating local endothelial function *in vivo* requires invasive methods, posing ethical issues.

On the other hand, numerous confounding factors affecting endothelial function make it difficult to provide direct causal evidence *in vivo*. To address these challenges, computational fluid dynamics (CFD) research offers a practical approach to exploring the relationship between morphology and endothelial dysfunction. CFD studies simulate blood flow conditions, calculate shear stress applied to the endothelial cell surface, and assess the impact of hemodynamics on endothelial cell function. CFD-calculated LAA wall shear stress magnitude and shear stress oscillation coefficient are related to stroke risk (Pons et al., 2022). Parameters derived from CFD-calculated shear stress correlate highly with myocardial fibrosis regions shown by cardiac MRI (Paliwal et al., 2021). Compared to general clinical studies, the advantage of CFD research is that it can isolate the LAA morphology as a single variable while keeping other parameters constant. Using CFD models, researchers have found that AF significantly reduces blood flow velocity within the LAA and decreases the rate of change of LAA wall shear stress, with the extent of reduction independent of LAA overall morphology classification (cauliflower-type, chicken wing-type, windbag-type, and cactus-type). Instead, local angles and lobar features of the LAA are decisive factors for the rate of change in wall shear stress (Musotto et al., 2022, 2024).

Additionally, different fusion methods of LAA models with simulated atria have shown that the relative position of the LAA to the LA significantly affects the blood flow state within the LAA, including LAA wall shear stress (Fang et al., 2021). Although CFD research builds a bridge to explore the relationship between morphology and endothelial function, it still has limitations. Current CFD models are based on multiple assumptions, such as blood viscosity, initial flow field conditions, and the rigid morphology of the LA and LAA. Variations in assumptions can lead to significant deviations in CFD results, so careful interpretation of CFD findings is necessary before developing biomimetic CFD models (Paliwal et al., 2021; García-Villalba et al., 2021). In addition to CFD research, our previous studies have found that in AF patients, compared to non-cauliflower-type LAA, cauliflower-type LAA has higher local von Willebrand factor, interleukin-6, and plasminogen activator inhibitor-1, and lower nitric oxide content, supporting the correlation between LAA morphology and local endothelial function (Xu et al., 2021).

In summary, AF is associated with localized endothelial dysfunction in the LAA. The LAA's morphology may affect local endothelial function through changes in hemodynamics.

#### Atrial cardiomyopathy

Atrial cardiomyopathy refers to a myocardial disorder characterized by morphological changes, mechanical dysfunction, and electrophysiological alterations of the atria, leading to clinically relevant manifestations. Based on the primary pathological features, it can be classified into cardiomyocyte type, fibroblast type, mixed cardiomyocyte and fibroblast type, and non-collagen deposits type (D'Alessandro et al., 2022). On the one hand, atrial cardiomyopathy can increase the risk of thromboembolism due to AF; on the other hand, atrial cardiomyopathy may itself be a risk factor for thromboembolic events. Atrial fibrosis is one of the most significant features of atrial cardiomyopathy. Compared to other types of stroke, patients with cryptogenic embolic stroke have significantly increased LA fibrosis (Fonseca et al., 2018). Pathological results from surgically removed LAA show a close correlation between LAA fibrosis and thrombus formation (Maher et al., 2023). In addition to atrial fibrosis, atrial cardiomyopathy is associated with mechanisms such as atrial amyloidosis, fat deposition, inflammatory cell activation, and endothelial dysfunction, which promote thrombosis (Tubeeckx et al., 2024; D'Alessandro et al., 2022). Although the clinical importance of atrial cardiomyopathy is evident, its diagnosis remains challenging and largely relies on imaging studies, especially cardiac MRI. However, due to the time-consuming nature of cardiac MRI, its widespread use is limited. Therefore, echocardiography and CT for evaluating atrial morphology and function present more clinically applicable diagnostic indicators. The size of the LA and LAA, the LA sphericity index, the degree of LA asymmetry, and the shape of the LA roof are morphological parameters associated with atrial fibrosis and AF outcomes, potentially representing manifestations of atrial cardiomyopathy. Recently, comprehensive analysis of LA and LAA shapes using statistical shape modeling (SSM) has led researchers to develop new, more extensive, and objective methods for LA and LAA shape assessment. These methods may predict outcomes following AF catheter ablation better than traditional shape parameters and potentially become new diagnostic indicators for atrial cardiomyopathy (Jia et al., 2021; Bieging et al., 2021). Moreover, they enable the computational assessment of comorbidities, allowing such conditions to be systematically studied through data-driven modeling.

### Hypercoagulable states

A substantial body of evidence confirms that patients with AF exhibit abnormalities in various anticoagulant and procoagulant factors. Recent reviews have summarized the correlations between these coagulation system markers and AF so this topic will be elaborated on here (Khan and Lip, 2019; Rafaqat et al., 2024). It is essential to note that most studies have only assessed the relationship between systemic blood markers and AF. To determine whether a hypercoagulable state exists locally in the LAA of AF patients, our team's previous research found significant differences in levels of plasminogen activator inhibitor-1, von Willebrand factor, interleukin-6, and platelet activation ratio in blood samples from the LA or LAA of AF patients compared to peripheral blood samples, but no “centripetal” distribution pattern for fibrinogen, D-dimer, and thrombin-antithrombin complex levels (Xu et al., 2021). This phenomenon might occur because most patients in our study were on long-term oral anticoagulant therapy, and prolonged AF episodes might lead to a gradual equilibration of blood marker levels between the intracardiac and peripheral blood. Bartus et al. found that even when there are no significant differences in blood marker concentrations, thrombus tests on samples from different sources show that thrombi formed from LAA samples are denser and require a longer time for fibrinolysis than those from peripheral blood (Bartus et al., 2020). Assessing a hypercoagulable state based solely on blood marker concentrations may not be comprehensive; thrombus characteristics and other evaluations are also necessary. Currently, there is a lack of studies on the relationship between LAA morphology and local hypercoagulable states. As mentioned, hemodynamic factors may influence the configuration and activity of coagulation factors, so theoretically, the morphology of the LAA could impact local hypercoagulability. Our previous research found that AF patients with a cauliflower-type LAA had a higher local platelet activation ratio than those with non-cauliflower LAA. At the same time, thrombin-antithrombin complexes, D-dimer, and fibrinogen levels were not associated with LAA morphology (Xu et al., 2021). Similarly, Kosiuk et al. found that platelet activation within the LAA was primarily related to LAA size rather than its morphological classification (Kosiuk et al., 2019).

In summary, AF patients are in a hypercoagulable state, with systemic comorbidities potentially being a significant factor. AF itself may contribute to the formation of local hypercoagulability. More evidence is needed regarding whether the morphology of the LAA or LA is related to local hypercoagulable states, and research is needed in this area.

## LAA morphology and risk of LAAT

Numerous studies have confirmed the association between the morphology of the LAA and stroke, as detailed in other reviews (Fang et al., 2022; Sun et al., 2023). However, studies examining the relationship between LAA morphology and LAAT are relatively limited, as summarized in Table 2. Unlike stroke and systemic embolic events, LAAT is asymptomatic and commonly diagnosed through invasive techniques such as TEE and CT scans, which involve X-ray radiation, making prospective studies challenging. All the studies listed in Table 2 are single-center, retrospective analyses. These studies predominantly include patients who underwent AF cardioversion or catheter ablation, leading to significant selection bias. Therefore, caution is required when interpreting these study results.

**Table 2:** Study on the correlation between LA and LAA morphology and the risk of thrombus formation in the LAA.

**Parameter**	**Researchers (publication year)**	Number of cases^*^	**CHA_2_DS_2_-VASc**	**Imaging technique**	**Result**
LAA Volume	Rafaqat et al. (2024)	28/444	1.8 ± 1.3	TEE	An LAA diastolic volume > 8.6 ml increases the risk of LAAT by 6 times.
LAA Orifice Area	Bieging et al. (2021)	26/149	3.1 ± 1.9	TEE	An LAA orifice area > 4.09 cm^2^ increases the risk of LAAT/SEC by 1.6 times.
	Khan and Lip (2019)	61/122	4.3 ± 0.3	TEE	LAA orifice area is inversely related to LAAT risk (OR = 0.98 per 1 mm^2^ increase).
LAA Orifice Diameter	Rafaqat et al. (2024)	28/444	1.8 ± 1.3	TEE	The maximum diameter of the LAA orifice is inversely related to LAAT risk (OR = 0.26 per 1 cm increase).
	Musotto et al. (2024)	213/2,591	≥2 points 64.8%	TEE	Each 1 mm increase in LAA orifice diameter increases the risk of LAAT/SEC by 24%.
LAA Depth	Rafaqat et al. (2024)	28/444	1.8 ± 1.3	TEE	Each 1 cm increase in LAA depth raises the risk of LAAT by 1.7 times.
LAA Lobes	He et al. (2020)	36/564	NA	TEE	Each additional lobe in the LAA increases the risk of LAAT by 1.5 times.
	Taina et al. (2014)	80/472	2.9 ± 1.8	CTA	Each additional lobe in the LAA increases the risk of LAAT/SEC by 1.4 times.
	Kosiuk et al. (2019)	46/336	≥2 points 51.4%	CTA	The risk of LAAT in patients with lobulated LAA is 3.2 times higher compared to those with non-lobulated LAA.
LAA Morphology Classification	Rafaqat et al. (2024)	28/444	1.8 ± 1.3	TEE	The risk of LAAT in patients with cauliflower-type LAA is 10.2 times higher than in those with chicken-wing-type LAA.
	Bartus et al. (2020)	102/306	Median (IQR): 4 (2, 5)	TEE	The risk of LAAT in patients with cauliflower-type and wind-sock-type LAA is 6.6 times and 4 times higher, respectively, compared to those with chicken-wing-type LAA.
Relative Position of LA and LAA	Lei et al. (2022)	42/323	2.7 ± 1.7	CTA	When the upper edge of the LAA orifice is higher than the upper edge of the left upper pulmonary vein orifice, the risk of LAAT increases by 7.6 times.

BSA, Body Surface Area; CTA, Computed Tomography Angiography; LA, Left Atrium; LAA, Left Atrial Appendage; LAAT, Left Atrial Appendage Thrombus; LAV, Left Atrium Volume; OR, Odds Ratio; SEC, Spontaneous Echogenicity Contrast; TEE, Transesophageal Echocardiography; TTE, Transthoracic Echocardiography.

^*^Number of cases with positive left atrial appendage thrombus/Total number of cases.

The findings from Table 2 indicate that indicators reflecting LAA size, including volume, LAA orifice diameter, LAA orifice area, and LAA depth, are the most common morphological parameters associated with LAAT. Notably, the correlation between LAA orifice size and LAAT remains contentious (Wang et al., 2023; Miki et al., 2022; Kiskaddon and Decker, 2023; Chen et al., 2017). This phenomenon may occur because LAA orifice size results from LA and LAA remodeling, which is positively correlated with the severity of remodeling and, consequently, with the risk of LAAT. Conversely, an enlarged LAA orifice may facilitate blood exchange between the LAA and LA, potentially alleviating blood stasis and acting as a protective factor against LAAT formation.

In addition to LAA size parameters, another category of LAAT-related parameters can be summarized as the complexity of the LAA, including LAA morphological classification, the number of LAA lobes, and the angle of the pectinate muscles (错误!未找到引用源。). As mentioned, the anatomical morphology of the LAA is highly variable, and these parameters are defined based on specific LAA characteristics, making it challenging to evaluate LAA comprehensively. Moreover, these parameters can be highly subjective; for instance, definitions of LAA morphological classification vary across studies (Chen et al., 2017; Suehiro et al., 2021; He et al., 2020). Even with standardized definitions, there are significant inter- and intra-researcher variations in LAA morphological classification and lobe count (Taina et al., 2014). Despite these limitations, research summarized in Table 2 generally suggests that more complex LAA morphology correlates with a higher risk of LAAT, such as cauliflower-shaped LAA, increased lobation, and larger pectinate muscle angles relative to the main lobe of the LAA. Recently, Lei and colleagues employed fractal analysis to quantitatively assess the complexity of LAA morphology based on surface curvature and roughness—referred to as fractal dimension—and found it to be significantly correlated with LAAT and stroke, with predictive power superior to the CHA2DS2-VASc score (Lei et al., 2022). Although the fractal dimension provides a more objective and comprehensive assessment of LAA morphology compared to previous parameters, it only evaluates the complexity of LAA morphology, not its overall shape.

Additionally, this study only assessed the fractal dimension, overlooking the role of the LA, while the interaction between the LA and LAA is an essential factor in LAAT formation (Fang et al., 2021). Recently, SSM has gained wide application and achieved significant results in the medical field (Bonsel et al., 2022; Corral Acero et al., 2022). SSM constructs shape space vectors through a series of landmark points, mathematically describing shapes, thereby converting shape data into statistical models. This method offers a comprehensive, objective, and quantitative description of shape. Bieging and colleagues utilized cardiac MRI data to create an SSM for the LA and LAA, enhancing stroke prediction capabilities when combined with the CHA2DS2-VASc score (Bieging et al., 2021). Therefore, SSM holds promise as a new method for comprehensive and objective evaluation of the overall morphology of the LA and LAA, offering new insights into the role of LA and LAA morphology in LAAT formation. The LAA is the primary source for thromboembolism in AF. The morphology of the LAA plays a significant role in blood stasis, morphological changes, and hypercoagulable states, which are directly or indirectly related to thrombus formation. Accurate evaluation of the morphology of the LAA can assist with risk stratification in patients with AF. Currently, the commonly used LAA morphological evaluation indicators are insufficiently comprehensive and objective. Recently, new imaging protocols have allowed for LA morphological remodeling and fibrosis assessment, which has been demonstrated to correlate with assessing the individual's risks of thromboembolic events and the practical imaging of patients with LAAT.

## Expert opinion

### Real-world impact and applicability

Left atrial appendage (LAA) anatomy is closely linked to thromboembolic risk in atrial fibrillation (AF), with over 90% of atrial clots originating from the LAA. Anatomical classifications have revealed that simpler shapes are associated with lower stroke rates, while more complex configurations correlate with higher risk. These insights suggest that LAA morphology may serve as an independent risk marker, augmenting existing clinical scores like CHA_2_DS_2_-VASc.

Clinically, such information could support more refined risk stratification. For patients at borderline risk, identifying a higher-risk LAA structure might prompt earlier anticoagulation or consideration of device-based closure. However, current guidelines do not include LAA shape in management algorithms, and widespread implementation faces obstacles: imaging is not routinely performed in all AF patients, and methods such as transesophageal echocardiography (TEE) are semi-invasive. Moreover, most current data derive from selective patient groups undergoing procedures, limiting generalizability. These challenges highlight the need for standardized protocols and broader validation before integration into practice.

### Areas for improvement and limitations

To apply these findings clinically, several issues must be addressed. First is the lack of standardization in LAA morphology assessment. Descriptive terms are prone to interobserver variability, and there's a need for objective, quantitative metrics—such as curvature, volume, or orifice dimensions—that reduce subjectivity.

Technical limitations in imaging also pose barriers. TEE is invasive, while cardiac CT and MRI, though more acceptable, require contrast or specialized equipment. Innovations like low-dose CT protocols, radiomics, and automated analysis may enhance feasibility. Functional evaluation (e.g., flow velocity, spontaneous echo contrast) may further enrich morphological data.

In research, the primary limitation remains the retrospective nature of most studies. Prospective trials are difficult due to the asymptomatic nature of LAA thrombus and the logistical burden of imaging large populations. Nonetheless, combining improved imaging techniques with clearer morphological criteria could advance both research and clinical application.

### Research potential and endpoints

Future research should validate whether LAA structure independently predicts thromboembolic events and improves risk models beyond existing scores. Prospective cohorts with long-term follow-up can establish if anatomical features correlate with incident stroke or thrombus formation, particularly in patients with otherwise moderate clinical risk.

Functional–structural correlations also merit attention. For instance, combining morphology with metrics such as LAA emptying velocity or atrial strain could elucidate underlying mechanisms of thrombogenesis. Computational modeling may help simulate hemodynamic consequences of varying shapes, identifying flow thresholds linked to clot risk.

In parallel, integrating machine learning could automate classification and prediction, enabling broader clinical use. Ultimately, randomized studies may explore whether patients with high-risk anatomy benefit from intensified anticoagulation or earlier mechanical intervention.

### Future direction of research in this area

While LAA shape remains a key focus, future research will likely situate it within the broader concept of atrial cardiomyopathy. Since LAA structure reflects underlying atrial remodeling and fibrosis, combining anatomical data with imaging markers of atrial health—such as LA volume, delayed enhancement, or voltage mapping—may yield a more holistic risk profile.

Therapeutically, the emphasis may shift from prediction to prevention. For instance, anti-inflammatory or structural-modifying therapies might reduce risk by improving atrial substrate. Likewise, device therapy will continue evolving, with future LAA occlusion strategies potentially tailored to specific anatomies.

Emerging approaches also explore early stroke risk markers in patients without documented AF, where LAA features could serve as part of preclinical risk identification. Thus, LAA morphology will likely form one component of a multifactorial precision strategy.

### Outlook for clinical adoption in 5–10 years

In the next five years, LAA morphology may transition from observational interest to a tool with clinical utility. If ongoing studies confirm predictive value, imaging could be recommended for selected AF patients, particularly those with intermediate CHA_2_DS_2_-VASc scores, to refine management decisions.

Advances in imaging software and artificial intelligence may allow quick, automated LAA characterization during standard workups. Combined with clearer evidence, this could lead to guideline updates incorporating anatomical risk into stroke prevention pathways. Broader use of occlusion devices may follow in patients with unfavorable anatomy and contraindications to long-term anticoagulation.

Multidisciplinary collaboration—including cardiology, neurology, radiology, and data science—will be essential to integrate anatomy, function, and outcomes into practice. By 2030, a more personalized AF management paradigm may emerge, in which anatomical features help guide prevention strategies alongside traditional clinical factors.
